# Non-Invasive Imaging Including Line-Field Confocal Optical Coherence Tomography (LC-OCT) for Diagnosis of Cutaneous Lymphomas

**DOI:** 10.3390/cancers16213608

**Published:** 2024-10-25

**Authors:** Martina D’Onghia, Maria Mendonça-Sanches, Maria Erasti, Alessandra Cartocci, Laura Calabrese, Azzurra Sirchio, Linda Tognetti, Anastasia Batsikosta, Stefano Lazzi, Mariano Suppa, Simone Soglia, Josep Malvehy, Javiera Perez-Anker, Emanuele Cencini, Alberto Fabbri, Pietro Rubegni, Elisa Cinotti

**Affiliations:** 1 Dermatology Unit, Department of Medical, Surgical and Neurological Sciences, University of Siena, 51300 Siena, Italy; martina.donghia@gmail.com (M.D.); maria.erasti@libero.it (M.E.); alessandra.cartocci@unisi.it (A.C.); laura.calabrese@unisi.it (L.C.); a.sirchio@student.unisi.it (A.S.);; 2 Dermatology Unit, Champalimaud Clinical Centre, Champalimaud Foundation, 1400-038 Lisbon, Portugal; mariasanxes@gmail.com; 3 Department of Medical Biotechnology, Pathological Anatomy Section, University of Siena, 51300 Siena, Italylazzi2@unisi.it (S.L.); 4 Department of Dermatology, Hôpital Erasme, Université Libre de Bruxelles, 1050 Bruxelles, Belgium; drmarianosuppa@gmail.com; 5 Dermatology Department, University of Brescia, ASST Spedali Civili di Brescia, 25123 Brescia, Italy; s.soglia@unibs.it; 6 Melanoma Unit, Hospital Clinic Barcelona, University of Barcelona, 08007 Barcelona, Spainjaviperezanker@gmail.com (J.P.-A.); 7 CIBER de Enfermedades Raras, Instituto de Salud Carlos III, 08034 Barcelona, Spain; 8 Department of Medical, Surgical and Neurological Sciences, Hematology Section, University of Siena, 53100 Siena, Italy; emanuele.cencini@ao-siena.toscana.it (E.C.); a.fabbri@ao-siena.toscana.it (A.F.)

**Keywords:** primary cutaneous lymphoma, skin, dermoscopy, LC-OCT, optical coherence tomography, RCM

## Abstract

**Background/Objectives:** Primary cutaneous lymphomas (PCL) are a heterogeneous group of non-Hodgkin lymphomas arising from malignant T (CTCL) or B (CBCL) cells, often mimicking other skin conditions. Recently, non-invasive diagnostic imaging modalities, including dermoscopy, Reflectance Confocal Microscopy (RCM), and Line-field Optical Coherence Tomography (LC-OCT), have become increasingly important, supporting clinicians in clinical practice. Hence, our study aimed to describe dermoscopic, RCM, and LC-OCT features of PCL and to explore their role in PCL management. **Methods**: Between December 2022 and January 2024, 40 lesions of 25 patients with PCL were retrospectively analyzed at the Dermatologic Unit of the University of Siena, Italy. Predefined dermoscopic, LC-OCT, and RCM criteria were assessed and their frequencies were calculated. **Results:** At dermoscopy, CTCL lesions were characterized by pinkish structureless areas (58,6%) and homogeneous distributed dotted vessels (35,7%), whereas 57.1% of CBCL presented with orange-yellow structureless areas. Considering CTCL, lymphocytes in the epidermis, dermal-epidermal junction, and dermis were detected by LC-OCT in 73.1%, 66.7%, and 51.9% and by RCM in 72.2%, 55.6%, and 61.1% of cases, respectively. The detection of lymphocytes was more precise using RCM than LC-OCT in CTCL (*p* < 0.001). Dermal infiltration of medium-reflective cells was visible in 80% and 40% of CBCL cases by LC-OCT and RCM, respectively. **Conclusions**: Non-invasive imaging techniques may support clinicians in managing PCL; however, further studies are mandatory in this field.

## Simple Summary

This study focused on the potential role of non-invasive imaging diagnostic techniques, including LC-OCT, in the diagnosis of primary cutaneous lymphomas. Data from 40 lesions in 25 patients with a histological diagnosis of primary cutaneous lymphomas were retrospectively evaluated. Considering cutaneous T-cell lymphomas, RCM proved to be more effective than LC-OCT in detecting lymphocytes in the epidermis and dermis. As for B cell lymphomas, (25% of cases) mostly presented as nodules, with medium-reflective cell infiltration detected in 80% of cases by LC-OCT. This study suggests that non-invasive imaging could be valuable in supporting the diagnosis of primary cutaneous lymphomas, though further research is needed.

## 1. Introduction

Primary cutaneous lymphomas (PCL) comprise a heterogeneous group of non-Hodgkin lymphomas (NHL), which range from indolent to aggressive lymphoproliferative disorders. By definition, these entities are confined to the skin at the time of diagnosis without extracutaneous involvement. Despite their rarity, they are the second most prevalent form of extranodal lymphomas [1,2]. In contrast to nodal NHL, most of which originate from B cells, cutaneous T-cell lymphomas (CTCL) comprise about 75–80% of all PCL, with two-thirds of them classified as mycosis fungoides (MF) or Sézary Syndrome (SS) [3,4,5]. The skin manifestations of early-stage MF include non-specific scaly patches and plaques, while advanced stages develop disseminated lesions, tumors, and erythroderma [6,7,8]. 

Cutaneous B-cell lymphomas (CBCL) encompass various subtypes with distinct clinical behaviors. Some, such as primary cutaneous marginal zone lymphoma (PCMZL) and primary cutaneous follicle center lymphoma (PCFCL), exhibit indolent characteristics, while others, including diffuse large B-cell lymphoma leg type (DLBCL-LT) and intravascular large B-cell lymphoma (IVLBCL), demonstrate aggressive behavior [9,10]. Consequently, they present heterogeneous clinical manifestations; thus clinicopathological correlation is essential for accurate diagnosis, proper treatment modalities, and prognostic outcomes [11]. 

Due to morphological and histological similarities of PCL with many inflammatory skin conditions, the diagnosis can be challenging even for experienced clinicians, taking around 36 months on average (ranging from 12 to 90 months) [12,13]. However, early diagnosis is crucial, as it can modify the disease course, avoiding unsafe treatments and minimizing disease progression [14,15]. On this background, a multidisciplinary approach is imperative for the appropriate management of patients with PCL [16]. 

Recently, although histopathology continues to be the gold standard for PCL diagnosis [17], the potential use of new non-invasive diagnostic imaging tools in this field, including dermoscopy, reflectance confocal microscopy (RCM), and line-field confocal optical coherence tomography line (LC-OCT), has gained increasing attention [18]. 

While the role of dermoscopy in PCL management has been extensively described [19], RCM features have been primarily characterized for CTCL, and little is known about the possible use of LC-OCT [20]. Particularly, LC-OCT has become increasingly used in clinical practice due to its ability to provide real-time 3D visualization of skin images in both vertical and horizontal view [21]. Indeed, this technology combines the strengths of OCT and RCM, effectively addressing their limitations in spatial resolution, penetration, and image orientation [22]. In addition, LC-OCT provides in vivo images of entire skin lesions at a cellular resolution similar to that of histological examination [23,24]. 

Hence, the goal of our study was to assess the dermoscopic, RCM, and LC-OCT characteristics of PCL and to explore the potential role of new non-invasive technologies in diagnosing these conditions.

## 2. Materials and Methods

This retrospective observational monocentric study was conducted using data from the Dermatology Department of the University of Siena (Italy). In total, 40 sites from 25 patients with histopathologically confirmed PCL diagnoses were consecutively selected between September 2021 and January 2024. All images were acquired by an expert in skin imaging with more than 10 years of experience in dermoscopy, RCM, and LC-OCT (E.C.).

The LC-OCT (DAMAE Medical, Paris, France) device consisted of a portable probe connected to a central unit and display, offering an acquisition rate of 10 frames/s, an axial resolution of 1.2 μm, a lateral resolution of 1.3 μm, a scanning depth of 500 μm, and a lateral field of view of 1.2 mm. To ensure refractive index matching, a drop of paraffin oil was applied between the lesion and the LC-OCT camera lens. Vertical sections, 3D images, and real-life videos were acquired during the examination.

In vivo, RCM examination was performed using a handheld VivaScope 3000 camera (MAVIG GmbH, Munich, Germany), which uses an 830-nm wavelength laser and provides images with a lateral resolution of 1 μm and axial resolution of 3−5 μm that correspond to a horizontal 920 μm × 920 μm section of the skin up to 250 μm in depth. A digital camera (Vivacam; Mavig, Germany) connected to an RCM computer was used to capture dermoscopic images when operating in RCM mode.

In our study, images were evaluated together by three dermatologists (M.D., M.S., and M.E.) with intermediate LC-OCT and RCM experience (about 12 months) and one expert dermatologist (E.C., >10 years of LC-OCT and RCM experience) who were blinded to any dermoscopic, LC-OCT, and RCM data. Any disagreement was resolved by consensus among the evaluators. At least three vertical and/or 3D LC-OCT images, one LC-OCT video, and at least one RCM image of different depths (epidermis, dermo-epidermal junction, and dermis) considered relevant were evaluated for each lesion.

According to the current literature, the following dermoscopic patterns were assessed under polarized dermoscopy: dotted vessels, linear vessels, linear curved vessels (spermatozoa-like structures), white scale, yellow scale/crusts, white structureless areas (patch blotches), orange-yellow structureless areas, bright red structureless areas, pinkish structureless areas, ulceration, and crystalline structures [19,25,26,27].

The RCM criteria were chosen based on previously described criteria suggestive of PCL features [28,29]. Based on our experience and prior proposed terms [20,27,28], for each RCM criterion, we assumed the presence of possible corresponding LC-OCT features (Table 1).

Descriptive statistics included the mean and standard deviation (SD) for quantitative variables, whereas frequency and percentage were reported for categorical variables. To compare RCM and LC-OCT features, a proportion test was performed. *p* < 0.05 was considered statistically significant. All analyses were performed using R software version 4.1.0 (R Foundation for Statistical Computing, Vienna, Austria).

## 3. Results

Overall, 40 lesions—10 (25%) CBCL and 30 (75%) CTCL—with a histopathologically confirmed diagnosis of PCL were included in this study. The lesions were found in 25 patients (40% female) (Supplementary Table S1). The mean (SD) age of the CTCL and CBCL cohort was 71 (12) years and 55 (15) years, respectively. Among mature T-cell and NK-cell lymphomas, the most prevalent lesions were MF (n = 19), followed by Sezary syndrome (SS) (n = 8), primary cutaneous aggressive epidermotropic CD8+ cytotoxic T-cell lymphoma (n = 2), and primary cutaneous anaplastic large cell lymphoma (PCALCL) (n = 1). CTCL lesions were primarily located on the lower legs, knees, and ankles (n = 8) and the arms and armpits (n = 8), followed by the back and shoulders (n = 3), thorax (n = 3), and abdomen (n = 3). Additionally, two lesions were located on the buttocks and thighs, and one lesion each on the feet, genital area, and scalp. Clinically, the majority of the lesions were patches (n = 19), followed by plaques (n = 9) and one nodule. 

Considering CBCL, 6 lesions were PCFCL, while 4 were PCMZL. CBCL lesions were mainly located on the scalp region (n = 7), followed by the lower leg (n = 1), the arm (n = 1), and the back (n = 1). Among the CBCL lesions, nine were nodules and one was a plaque.

Pinkish structureless areas (58.6%) were the most common CTCL dermoscopic pattern, followed by dotted vessels (35.7% uniform and 17.9% clustered) and white scales (31%). The most common dermoscopic features of CBCL were linear vessels (71%), orange structureless areas (57.1%), and bright red structureless areas (42.9%) (Table 2). 

Atypical epidermal architecture (50% vs. 61.6%), epidermotropism (73.1% vs. 72.2%), and lymphocyte infiltration at the dermo-epidermal junction (66.7% and 55.6%) and in the dermis (51.9% and 61.1%) were found in most CTCL evaluations under both LC-OCT and RCM, respectively (Table 3). 

Pautrier’s microabscesses (22.3% vs. 3.8%), spongiosis (22.2% vs. 7.7%), and dendritic cells (27.5% vs. 3.8%) were most commonly observed under RCM evaluation compared to LC-OCT; while erosion and ulceration in the dermis were exclusively observed through LC-OCT and were detected in 11.1% of cases.

Considering the CBCL, telangiectasia (100% vs. 20%) and lymphocyte infiltration in the dermis (80% vs. 40%) were observed mainly under LC-OCT evaluation compared with RCM.

Overall, LC-OCT and RCM led to the exploration of the epidermis beneath the stratum corneum in the majority of the CTCL cases, with no statistically significant differences when comparing the two techniques (all *p* > 0.05). Instead, parakeratosis (*p* = 0.003), as well as hyperkeratosis (*p* < 0.001) and epidermal thickness (0.008), were found to a greater extent with LC-OCT than with RCM (see Table 3).

## 4. Discussion

Cutaneous lymphomas are a heterogeneous group of rare malignancies with a wide range of clinical presentations, posing a diagnostic challenge to dermatologists. The goal of our study was to describe the dermoscopic, RCM, and LC-OCT features of PCL in a series of CTCL and CBCL.

To date, dermoscopy represents the cornerstone of dermatologic diagnosis, enabling the visualization of sub-macroscopic structures in the epidermis and upper dermis, thus increasing the diagnostic accuracy of malignant and inflammatory dermatoses [30,31,32,33]. It has recently been described that PCL exhibits distinct and characteristic dermoscopic patterns that reflect specific lymphoma subtypes and their corresponding histopathological features, which are generally not observed in other inflammatory skin conditions. As for CTCL, Lallas et al. first described the distinct features of early-stage MF, a vascular structure resembling spermatozoa, emphasizing the significance of dermoscopy in differentiating it from chronic dermatitis [34]. More recently, Errichetti et al. [26] conducted a retrospective analysis of 118 MF lesions, defining distinctive dermoscopic profiles for each stage and variant of MF [26]. The authors demonstrated that linear-curved vessels and white vessels were significantly associated with patch-stage MF, while clustered dotted vessels were related to plaque-stage MF [26]. 

**Figure 1:**
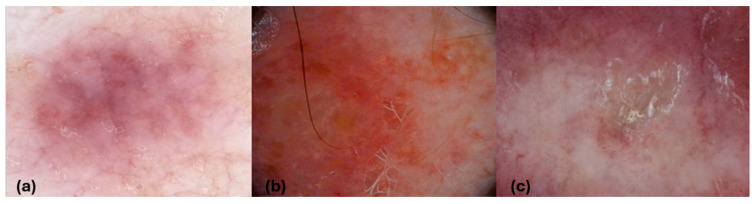
Mycosis fungoides (**a**,**c**), and PCFCL (**b**). Dermoscopic images (**a**–**c**). (**a**) Pinkish structureless areas; (**b**) orange-yellow structureless areas; (**c**) white scales, dotted clustered vessels, bright red structureless areas in the periphery, and a structureless area in the center of the lesion.

Our study partially corroborated previous findings regarding dermoscopic features of PCL. We found that the most common feature in CTCL lesions was the presence of structureless pinkish areas, observed in 58.6% of cases, which may be linked to dynamic changes in the cutaneous vasculature and suprapapillary network (Figure 1). 

Linear curved vessels (resembling spermatozoa-like structures) were predominantly seen in the patch stage and detected in 24% of cases. Additionally, uniformly distributed dotted vessels were the most frequently observed subtype, appearing in 35.7% of cases. Lastly, we noted white scales in 31% of cases, findings that may be attributed to the use of polarized dermoscopy, which enhances the visibility of scales.

As for CBCL, dermoscopic characteristics were initially elucidated by Mascolo et al., who demonstrated the efficacy of dermoscopy in augmenting PCL clinical diagnosis [35]. Subsequent research, which involved a blinded review of dermoscopic images of 58 CBCL lesions, identified two main encountered dermoscopic traits: a salmon-colored area/background and serpentine vessels [36]. Although these dermoscopic features lack specificity, their presence could indicate the risk of CBCL [32,37]. In our analysis, the most common dermoscopic features were linear vessels (71%), orange structureless areas (57.1%), and shiny white structures (42.9%). These features may correspond to the presence of linear vessels in the dermis, areas of erythrocyte extravasation, and dense lymphoid infiltrates that could be associated with dermal fibrosis.

RCM is a non-invasive technique that enables the in vivo visualization and capture of cellular-resolution images of skin lesions in parallel (en face) to the skin surface at various depths ranging from the stratum corneum to the papillary dermis [38]. This technique, which utilizes melanin and keratin as the primary endogenous chromophores, provides high, nearly histological resolution of skin structures. Primarily used for the diagnosis of melanocytic skin tumors, its current application also includes the differential diagnosis of inflammatory dermatosis [39,40,41]. 

Initially, the use of RCM for diagnosing CTCL, especially MF, was found to be unsatisfactory. The clinical applicability of RCM has been questioned due to its lack of cellular detail, which complicates the differentiation of cell types. This deficiency in detail impairs the ability to characterize the epidermotropic infiltrate as lymphocytic and to identify cytological atypia within the infiltrate [28,42].

However, RCM morphological criteria have been subsequently proposed for diagnosing erythematosquamous skin diseases, including early-stage MF, enhancing diagnostic reliability in this area [29,43]. Koller et al. indicated that interobserver agreement for diagnosing MF with RCM improves when epidermal atypical lymphocytes, interface dermatitis, pleomorphic tumor cells, and dendritic cells are present [44]. Additionally, the visualization of Pautrier abscesses by RCM appears to be linked with better histopathologic diagnosis and the detection of TCR gene clonality in the affected skin [45].

Conversely, the potential role of LC-OCT in diagnosing PCL has only recently been explored [33]. Soglia et al. clarified its application in MF through a study involving 10 patients. They highlighted that a combination of features, particularly atypical lymphocytes in the epidermis, dermo-epidermal junction, and dermis, could aid in diagnosing MF. Additionally, the identification of Pautrier’s microabscesses and a dense dermal inflammatory infiltrate was noted as stage-specific, making them valuable for monitoring pre-existing lesions during follow-up [33].

From our study, both RCM and LC-OCT have proven valuable for exploring the epidermis and dermo-epidermal junction in CTCL. Particularly, epidermotropism (73.1% vs. 72.2%) and lymphocyte infiltration at the dermo-epidermal junction (66.7% and 55.6%) were detected with both LC-OCT and RCM, respectively, in a high percentage of CTCL cases, with no statistical differences when comparing the two techniques (all *p* > 0.005). 

**Figure 2:**
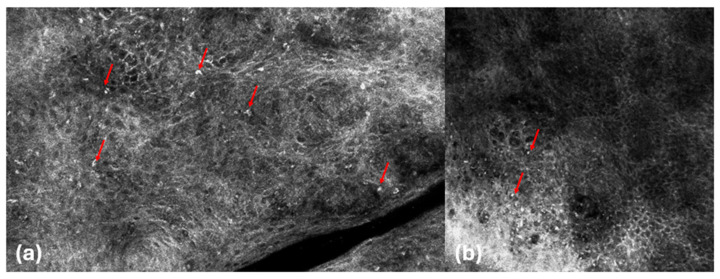
Mycosis fungoides. RCM images (**a**,**b**). Hyper-reflective round cells in the epidermis (red arrows).

Under both techniques, lymphocytes were visible as roundish hyper-reflective cells smaller than keratinocytes without visible nuclei inside, although it was not possible to clearly distinguish them from granulocytes (Figure 2). 

**Figure 3:**
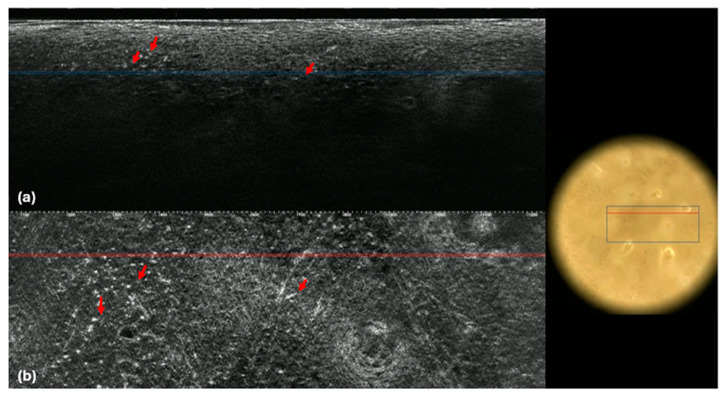
Mycosis fungoides. LC-OCT images (**a**,**b**). Vertical (**a**) and horizontal (**b**) sections showing roundish hyper-reflective cells in the dermis (red arrows).

Notably, lymphocytes were better defined under RCM than LC-OCT, resembling part of the hyper-reflective membrane of keratinocytes. From our experience, we noted that at LC-OCT, 3D and horizontal images can help better define the presence of these cells (Figure 3).

**Figure 4:**
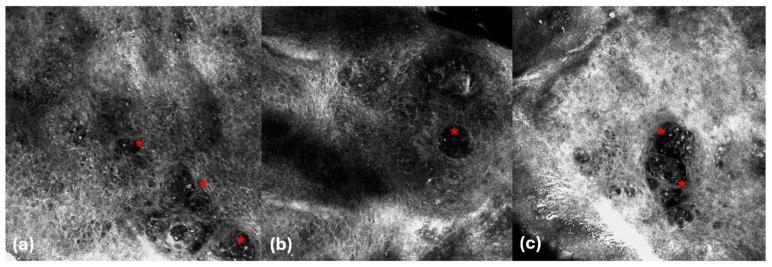
Mycosis fungoides. RCM images (**a**–**c**). Pautrier’s microabscesses in the epidermis (asterisks).

**Figure 5:**
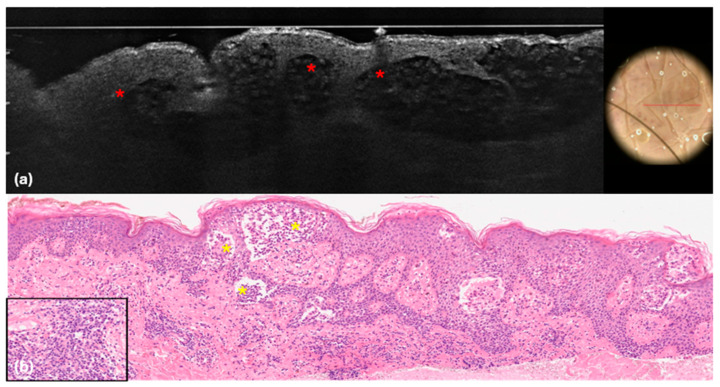
Mycosis fungoides. LC-OCT (**a**) and histopathological (**b**) images. (**a**) Hypo-reflective cavities with hyper-reflective particles inside (red asterisks); (**b**) epidermal hyperplasia with spongiosis, large vesicles, and lymphocytic epidermotropism (yellow asterisks). Dermal infiltrate of small to mid-sized T lymphocytes (insert). Haematoxylin and Eosin; Original magnification ×10; Insert, ×20.

As expected, DEJ attacks by lymphocytes were less visible under RCM because it is more difficult to define DEJ levels in horizontal images than in vertical and 3D LC-OCT images. Considering parakeratosis (51.9% vs. 5.6%, *p* = 0.003), as well as hyperkeratosis (84.6% vs. 11.1%; *p* < 0.001), these criteria were more visible in LC-OCT than RCM. It should be considered that LC-OCT allows the evaluation of a large area of the skin in a single image through vertical and horizontal sections, whereas RCM allows images only in the horizontal section and has a smaller field of view that can hamper the identification of parakeratosis or hyperkeratosis. In addition, the identification of parakeratosis is probably easier with LC-OCT than RCM because the cell nuclei within the stratum corneum are easily visible as hypo-reflective areas on LC-OCT, and the definition of parakeratosis on RCM remains controversial [46]. On the other hand, the atypical epidermal architecture was more detectable under RCM than under LC-OCT (61.6% vs. 50%; *p* = 0.008), probably because of the higher magnification of this technique compared with LC-OCT, which allows better identification of architectural alterations. Specifically, the atypical epidermis appeared as a variation in the shape and size of epidermal keratinocytes (irregular honeycomb pattern) in RCM and as a variation in the shape and size of the epidermal nuclei of keratinocytes and a variation in the reflectivity of keratinocytes in LC-OCT (figure). From our study, Pautrier’s microabscesses (22.3% vs. 3.8%) (Figure 4), spongiosis (22.2% vs. 7.7%) (Figure 5), and dendritic cells (27.5% vs. 3.8%) were visible in a minority of cases and were mainly identified by RCM than by LC-OCT. 

**Figure 6:**
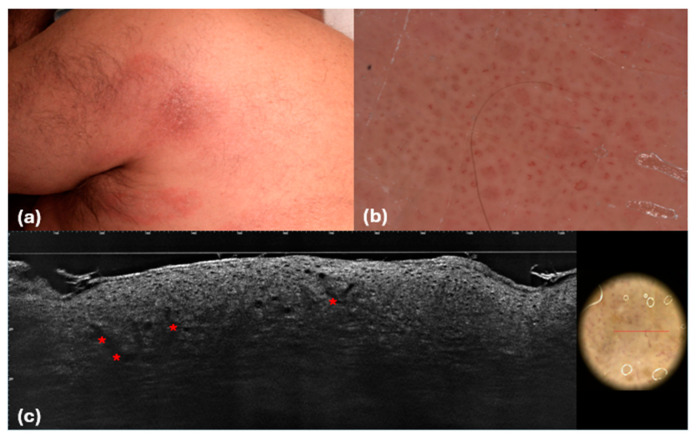
Mycosis fungoides, patch stage. Clinical (**a**), dermoscopic (**b**), and LC-OCT (**c**) images. (**b**) Dermoscopic imaging of the lesion reveals **a** short curved linear vessel similar to a spermatozoon (spermatozoa-like vessels), which corresponds at LC-OCT (**b**) to short glomerular vessels in the dermis.

As expected, blood vessels in the dermis were better visualized under LC-OCT than under RCM, given the wider field of view and deeper penetration of LC-OCT as previously stated (Figure 6). 

Lastly, lymphocyte dermal infiltration in CTCL was detected using both techniques, with a slightly higher prevalence noted with RCM (77.8% vs. 58.8%, *p* > 0.05). This difference is likely due to the difficulty of distinguishing hyper-reflective lymphocytes from surrounding hyper-reflective collagen in LC-OCT, coupled with RCM’s superior ability to visualize cytological details. 

The current literature on the use of RCM and LC-OCT for CBCL is limited. RCM’s depth penetration restricts its ability to describe features of these dermal proliferations. Meanwhile, LC-OCT, an emerging non-invasive imaging technique, has yet to be used for characterizing CBCL features.

In a recent clinical case report detailing relapsing secondary cutaneous follicular lymphoma with dermoscopy, RCM, and histology, the presence of nucleated cellular infiltration, notably surrounding appendages with associated fibrosis, proved to be particularly informative in the process of differential diagnosis against other pathological entities [27]. 

In our cases, CBCL primarily affects the medium and deep dermis and spares the epidermis, and tumor areas were visible in most cases using both LC-OCT and RCM. Additionally, lymphocyte infiltration in the dermis was more frequently detected with LC-OCT (80%) compared to RCM (40%). Notably, CBCL located on the scalp was better visible under non-invasive imaging techniques, possibly due to the underlying bone plate that facilitated the acquisition of images by providing a rigid underlying surface that did not cause the probes to sink and allowed the device not to lose its penetration capacity. 

**Figure 7:**
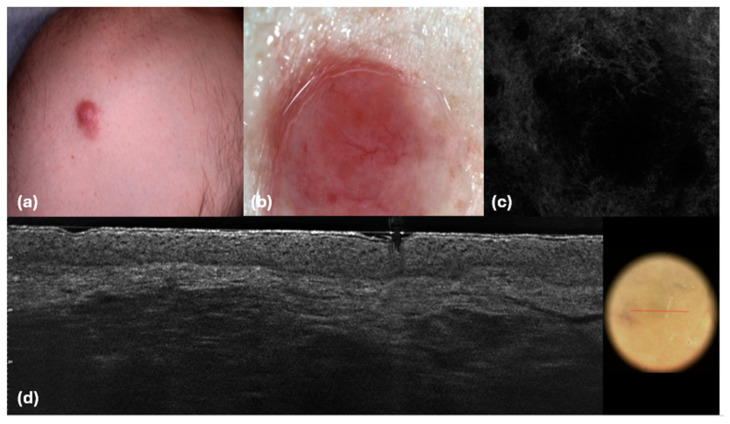
PCMZL presents as an isolated nodule. Clinical (**a**), dermoscopic (**b**), RCM (**c**), and (**d**) LC-OCT images. (**b**) Bright red structureless areas and linear vessels with branches; (**c**) Hypo-reflective well-defined large dark areas in the dermis with small hyper-reflective linear traits detached from the epidermis; (**d**) Hypo-reflective well-defined large dark areas in the dermis with small hyper-reflective linear traits and with hyper-reflective small cells inside detached from the epidermis; the epidermis is spared.

**Figure 8:**
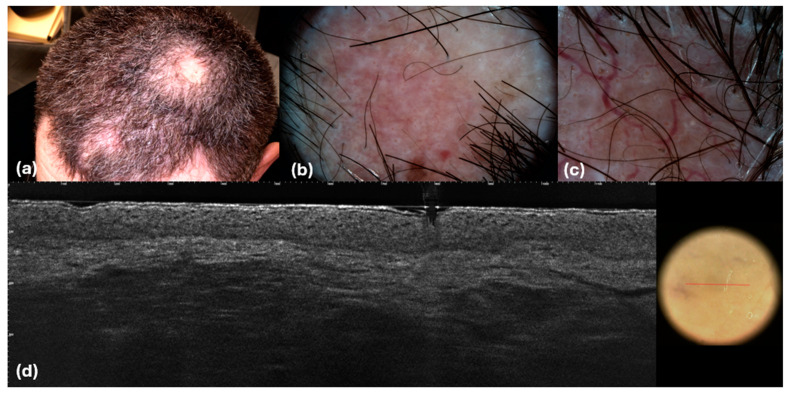
PCFCL presents as an isolated nodule. Clinical (**a**), dermoscopic (**b**,**c**), and LC-OCT (**d**) images. (**b**) Pinkish structureless areas; (**c**) linear vessels with branches; (**d**) hypo-reflective well-defined large dark areas in the dermis with hyper-reflective small cells inside; the epidermis is spared.

PCFCL and PCMZL appeared under LC-OCT and RCM as hypo-reflective well-defined large areas in the dermis with medium-reflective small and medium-sized cells inside, while the epidermis was spared with a Grenz zone of bright collagen in all cases (Figure 7 and Figure 8). 

**Figure 9:**
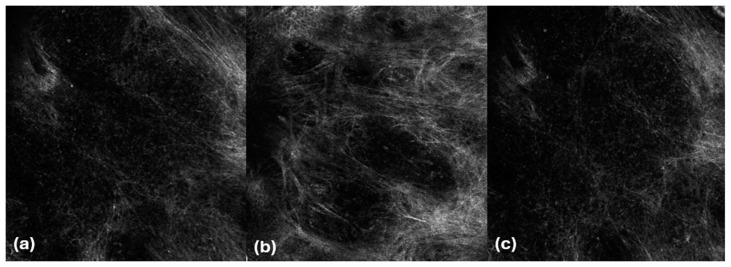
PCFCL. RCM images (**a**–**c**). Hypo-reflective roundish areas inside the dermis contain small, medium-reflective roundish B cells, separated by fine collagen septa.

All these aspects were clearly more visible in real-time images, 3D images, and videos, particularly cell infiltration. PCFCL were demarcated by surrounding collagen and formed multiple well-defined oval areas detached from the epidermis, particularly visible under RCM as they were surrounded by fine collagen septs (Figure 9). 

Telangiectasia was also found in CBCL evaluation, with a higher prevalence under LC-OCT (100%) than under RCM (20%), possibly due to the higher penetration of LC-OCT.

**Figure 10:**
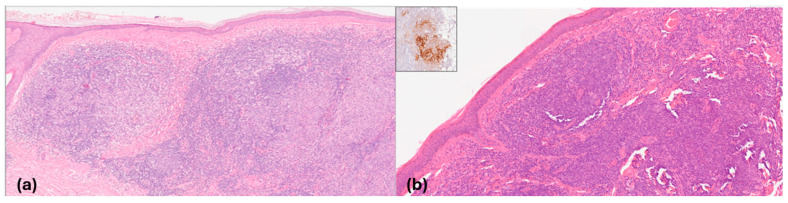
PCFCL (**a**) and PCMZL (**b**) lymphomas. Histological images (**a**,**b**). (**a**) Dense vaguely nodular infiltrates within the entire dermis that extend into the subcutaneous fat. The epidermis is spared. Haematoxylin and Eosin; original magnification (OM): A, ×1; B, ×4; C, ×10; D, ×20; (**b**) nodular infiltrate with residual germinal center colonized (insert, CD21) and diffuse small to mid-size B cell infiltrate in the dermis with a conspicuous T cell component. The epidermis is spared with a Grenz zone. Haematoxylin and Eosin; original magnification (OM): ×10. immunohistochemistry, chromogen diaminobenzidine (insert); original magnification (OM): CD21, ×10.

Although we did not identify specific characteristics of PCL in our LC-OCT and RCM evaluations, our findings partially correspond with their well-known histological architectural features (Figure 10).

Considering CBCL, they are histopathologically characterized by a nodular or dense cell infiltrate extending throughout the dermis and typically involving the subcutaneous tissues, as observed under RCM and LC-OCT [47,48]. In addition, through both techniques, the epidermis was spared as a rule [47,48]. Finally, we observed that PCFCL displayed more well-defined nodules compared to PCMZL, which were clearly visible under RCM. These nodules exhibited a distinct follicular pattern that matched the “back-to-back” follicular architecture seen in histologic findings [47].

Considered a bridge between dermoscopic morphology and histopathology, new non-invasive skin techniques have gained significant interest in the scientific community for their ability to enhance diagnostic accuracy, particularly in skin cancers, while their application in other dermatological fields, including inflammatory conditions, is still evolving. Specifically, those techniques have shown potential in highlighting microscopic features such as acanthosis, spongiosis, or vascular inflammation, allowing detailed analysis of inflammatory processes even in subclinical stages. The primary strength of this study is that we describe dermoscopic, RCM, and LC-OCT characteristics of PCL, showing the ability to observe tumor lymphocytes using both RCM and LC-OCT. These lymphocytes appeared as sparse hyper-reflective cells in CTCL and as aggregated medium-reflective cells in CBCL. Although these techniques are currently not sufficient to make a diagnosis, they could still provide valuable support for dermatologists in the diagnostic process. Moreover, they could also assist in selecting biopsy areas with a higher concentration of tumor cell infiltration or identifying tumor persistence after treatments. Additionally, they may also be useful for assessing disease activity within a lesion and assisting in the clinical management of patients with MF [42,49,50,51].

However, several limitations need to be recognized. The primary is the small sample size of patients, mainly due to the rarity of the disease. Another notable constraint concerns the retrospective design of this study, which is susceptible to recall and observation biases. Additionally, data from our study mainly included MF, SS, PCFCL, and PCMZL and did not account for the inherent heterogeneity of these lymphomas, which can result in varying morphological descriptions of the lesions and clinical stages. 

## 5. Conclusions

Our study detailed the dermoscopic, RCM, and LC-OCT features of PCL. Notably, RCM appears to detect lymphocytes in MF and SS more effectively than LC-OCT, while LC-OCT may be better suited for identifying CBCL, which is typically located deeper in the skin. However, further research on this topic is needed, and there is a clear unmet need for prospective, multicenter, controlled studies to assess the effectiveness of both established and emerging imaging techniques in this specialized field of dermato-oncology. 

## Figures and Tables

**Table 1:** Description of the selected RCM/LC-OCT criteria for PCL, based on the literature review and expert opinion.

CTCL
**Epidermis**
*Parakeratosis * Small hypo-reflective areas corresponding to cell nuclei are visible inside keratinocytes of the stratum corneum.
*Hyperkeratosis * Stratum corneum thicker than 20 μm. It is measured on a stack in RCM and on a vertical section in LC-OCT.
*Epidermal thickness* Variation in thickness of epidermis (normal; atrophy; acanthosis). It is measured on a stack in RCM and on a vertical section in LC-OCT.
*Epidermal architecture * Atypical epidermis: variation in shape and size of epidermal keratinocytes (irregular honeycomb pattern) in RCM and of nuclei of epidermal keratinocytes in LC-OCT (normal, atypical, disarray). Disarray: disarranged epidermis.
*Epidermotropism* Hyper-reflective round cells 5 to 10 μm in diameter inside the epidermis.
*Pautrier’s microabscesses * Hypo-reflective roundish areas in the epidermis with hyper-reflective small round cells inside.
*Spongiosis* Hypo-reflective areas among epidermal keratinocytes with hyper-reflective small round cells inside.
*Dendritic Cells* Hyper-reflective stellate cells.
*Erosion or ulceration* Hypo-reflective areas with sharp borders and irregular contours in the upper part of the skin, filled with amorphous material and cellular debris.
**DEJ**
*Interface dermatitis/non-edged papillae * Not defined DEJ.
*Junctional sparse lymphocytes * Hyper-reflective round cells 5 to 10 μm in diameter at the DEJ.
**Dermis**
*Blood vessel dilatation * Elongated large hypo-reflective structures with possible visible blood cell flow inside the dermis.
*Lymphocyte infiltration* Hyper-reflective round cells 5 to 10 μm in diameter in the dermis.
*Fibrosis* Increased number of refractile fibrous bundles in RCM and increased refractivity of the dermis in LC-OCT.
*Folliculotropism* Hyper-reflective round cells 5 to 10 μm in diameter around hair follicles
**CBCL**
*Blood vessel dilatation * Elongated large hypo-reflective structures with possible visible blood cell flow inside the dermis.
*Lymphocyte infiltration* Well-defined hypo-reflective areas filled with hyper-reflective round cells 5 to 10 μm in diameter inside of the dermis. At RCM these areas can be delineated by hyper-reflective collagen bundles.

**Legend**: **CBCL**, cutaneous B cell lymphomas**; DEJ**, dermo-epidermal junction; **PCL**, primary cutaneous lymphoma; C**TCL**, cutaneous T cell lymphomas.

**Table 2:** Distribution of specific dermatoscopic features of CTCL and CBCL across study lesions.

Dermatoscopic Features	CBCL (n = 7)	CTCL (n = 29)
**Vessels**		
Dotted		
Uniform, n (%)	0	10 (35.7)
Clustered, n (%)	0	5 (17.9)
Linear, n (%)	5 (71.4)	4 (13.8)
Linear vessels with branches, n (%)	2(28.5)	0
Linear curved (spermatozoa-like structures), n (%)	0	7 (24.1)
**White scale, n (%)**	1 (14.3)	9 (31)
**Yellow scale/crusts, n (%)**	0	2 (6.9)
**White structureless patches, n (%)**	1 (14.4)	3 (10.3)
**Orange-yellow structureless areas, n (%)**	4 (57.1)	0
**Bright red structureless areas, n (%)**	3 (42.9)	2(6.9)
**Pinkish structureless areas, n (%)**	2 (28.6)	17 (58.6)
**Ulceration, n (%)**	0	1 (3.4)
**Shiny white structures (crystalline structures or chrysalis), n (%)**	3 (42.9)	3 (10.3)

**Legend**: **CBCL**, cutaneous B cell lymphomas**; DEJ**, dermo-epidermal junction; **PCL**, primary cutaneous lymphoma; C**TCL**, cutaneous T cell lymphomas.

**Table 3:** Distribution and Agreement between LC-OCT and RCM criteria of PCL.

PCL Criteria	LC-OCT	RCM	*p*-Value
**CTCL**			
**Epidermis**			
Parakeratosis, n (%)	14 (51.9)	1 (5.6)	0.003
Hyperkeratosis, n (%)	22 (84.6)	2 (11.1)	<0.001
Epidermal thickness			
Normal, n (%)	8 (30.8)	17 (94.4)	Na
Atrophy, n (%)	9 (34.6)	1 (5.6)	
Acanthosis, n (%)	9 (34.6)	0 (0)	
Epidermal architecture			
Normal, n(%)	13 (50)	6 (33.3)	0.008
Atypical, n (%)	13 (50)	12 (66.6)	
Epidermotropism, n (%)	19 (73.1)	13 (72.2)	0.686
Pautrier’s microabscesses, n (%)	1 (3.8)	4 (22.3)	0.333
Spongiosis, n (%)	2 (7.7)	4 (22.2)	0.333
Dendritic Cells, n (%)	1 (3.8)	6 (37.5)	0.089
Erosion/Ulceration, n (%)	3 (11.1)	0	Na
**DEJ**			
Interface dermatitis/non-edged papillae, n (%)	7 (28)	3 (16.7)	Na
Junctional lymphocytes, n (%)	18 (66.7)	10 (55.6)	0.999
**Dermis**			
Blood vessels dilatation, n (%)	21 (77.8)	10 (58.8)	Na
Lymphocyte infiltration, n (%)	14 (51.9)	11 (61.1)	Na

**Legend**: **CBCL**, cutaneous B cell lymphomas; **DEJ**, dermo-epidermal junction; **PCL**, primary cutaneous lymphoma; C**TCL**, cutaneous T cell lymphomas.

## Data Availability

The data presented in this study are available on request from the corresponding author.

## Supplementary Materials

The following supporting information can be downloaded at: https://www.mdpi.com/article/10.3390/cancers16213608/s1. Table S1. Demographic and clinical features of PCL patients.
