# Supplementary material for: Non-Invasive Imaging Including Line-Field Confocal Optical Coherence Tomography (LC-OCT) for Diagnosis of Cutaneous Lymphomas
Source: Cancers (Basel). 2024 Oct 25;16(21):3608. doi: 10.3390/cancers16213608 (PMC11544893; doi:10.3390/cancers16213608)
Supplement: Supplementary file 1 [file cancers-16-03608-s001.zip › cancers-3218279-supplementary.pdf]

Supplementary Table S1. Demographic and clinical features of PCL patients.

	Sex	Age (years)	Histologic diagnosis	Number of sites examined
**TCL**				
Patient 1	M	75	Mycosis Fungoides	1
Patient 2	F	82	Mycosis Fungoides	1
Patient 3	M	82	Sezary Syndrome	1
Patient 4	M	66	Mycosis Fungoides	3
Patient 5	F	81	Mycosis Fungoides	2
Patient 6	F	84	Mycosis Fungoides	1
Patient 7	M	51	Mycosis Fungoides	1
Patient 8	M	71	Mycosis Fungoides	2
Patient 9	F	69	Mycosis Fungoides	3
Patient 10	M	30	Mycosis Fungoides	1
Patient 11	M	69	Mycosis Fungoides	1
Patient 12	F	55	Mycosis Fungoides	1
Patient 13	F	72	Sezary Syndrome	5
Patient 14	M	72	Mycosis Fungoides	1
Patient 15	M	62	Sezary Syndrome	1
Patient 16	F	70	Sezary Syndrome	1
Patient 17	M	94	Mycosis Fungoides	1
Patient 18	M	81	Mycosis Fungoides	3
**BCL**				
Patient 1	F	57	PCMZL	1
Patient 2	M	56	PCMZL	1
Patient 3	M	70	PCMZL	1
Patient 4	M	38	PCFCL	1
Patient 5	M	38	PCFCL	3
Patient 6	F	72	PCFCL	2
Patient 7	F	69	PCMZL	1

Legend: CBCL, cutaneous B cell lymphomas**;** C**TCL**, cutaneous T cell lymphomas; **PCFCL**, primary cutaneous follicle center lymphoma; **PCMZL**, primary cutaneous marginal zone lymphoma.
